# Subacromial impingement by a lipoma arborescens

**DOI:** 10.1051/sicotj/2021004

**Published:** 2021-03-11

**Authors:** Mohamed Elamin, Venkatramana Yeluri, Hisham Khatir, Paul O’Grady, Fadel Bennani

**Affiliations:** 1 Department of Orthopaedics, Mayo University Hospital Castlebar Co. Mayo F23 H529 Ireland; 2 Department of Pathology, Mayo University Hospital Castlebar Co. Mayo F23 H529 Ireland

**Keywords:** Shoulder impingement, Lipoma, Lipoma arborescens, MRI

## Abstract

Subacromial impingement syndrome (SIS) is the leading cause of shoulder pain. A systemic approach for abnormal causes of SIS is recommended to avoid misdiagnosing rare or sinister pathologies. To our knowledge, only nine cases of subacromial lipoma arborescens associated with impingement syndrome have been reported in the literature. In this report, we briefly discuss histopathologic and radiological signs of an unusual case of impingement syndrome caused by subacromial “lipoma arborescens” and describe arthroscopic synovectomy after the failure of conservative management. The patient remains symptom-free five years after surgery.

## Introduction

Subacromial impingement syndrome (SIS) is the leading cause of shoulder pain, representing 40–60% of all shoulder pain visits to orthopaedic clinics [1]. As an extrinsic cause of shoulder impingement [2], mechanical irritation of the subacromial bursa and rotator cuff, by the coracoacromial arch, is the most common cause of impingement syndrome [3]. Of these, there are few reported tumours arising from the coracoacromial arch [3, 4]. In addition, limited cases of sub-acromial space-occupying lesions have been reported as a cause of SIS, such as lipoma [5, 6] and lipoma arborescens [7–15].

Moreover, few lesions found outside the subacromial space such as supraspinatus intramuscular lipoma or cysts and suprscaular glomus tumour can also cause subacromial impingement [16]. When compared to shoulder disorders (e.g., subacromial impingement, rotator cuff disorders), tumours in the shoulder region should not be missed, as they are not uncommon [17]. These tumours include metastatic carcinoma and myeloma in the glenoid, chondrosarcoma, osteosarcoma, chondroblastoma, osteoid osteoma, plasmacytoma, lymphoma, giant cell tumour of the coracoid process [17–19].

To our knowledge, nine cases of subacromial lipoma arborescens associated with impingement syndrome have been reported in the literature [7–15].

We describe an unusual case of impingement syndrome caused by subacromial “lipoma arborescens” and treated with arthroscopic synovectomy following the failure of conservative management.

## Case report

A 55-year-old lady complained of left shoulder pain. She worked as a clerical officer, and found it difficult to do any heavy lifting, overhead work or to sleep at night. The pain started gradually over the last 10 months, she did not recall any trauma or injuries. She had no significant weight loss or constitutional symptoms, there were no red flags and her history was otherwise normal. On physical exam, she had painful impingement of her left shoulder with some weakness of her rotator cuff muscles. Neer and Hawkins signs were both positive. She had no lymphadenopathy and there was no evidence of any skin changes. Blood investigations were normal and plain films of the left shoulder were unremarkable.

**Figure 1:**
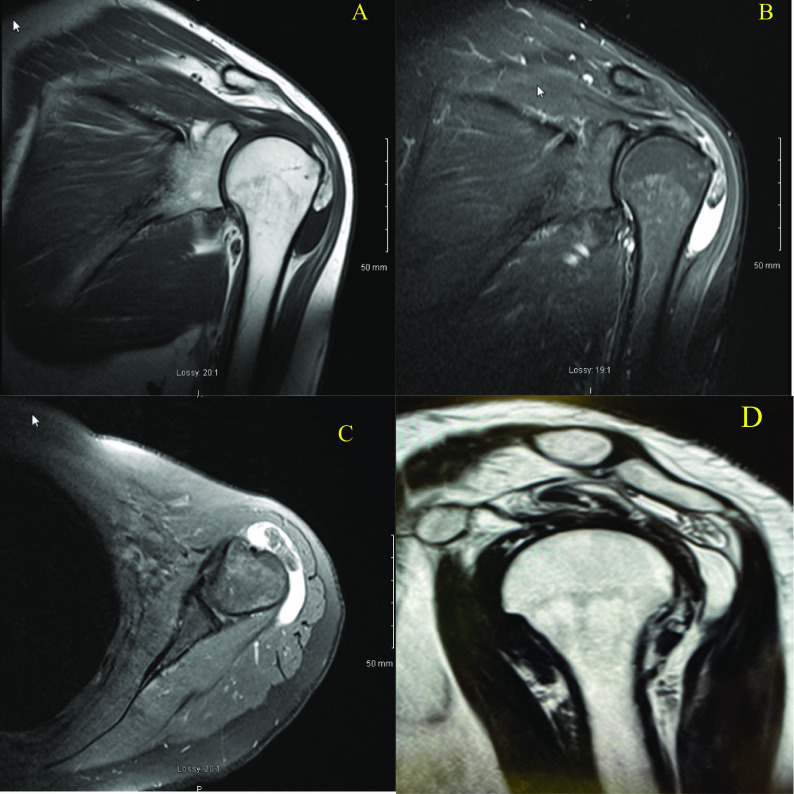
Extra-articular lipoma arborescens. (A) T1 coronal MRI (B) T2 coronal MRI (C) T2 axial MRI and (D) T1 sagittal MRI showing: fat-containing, lobulated synovial mass (2.5 × 1.0 × 0.5 cm) in the subacromial-subdeltoid bursa with fat signal intensity on all sequences.

She had temporal relief from a steroid injection and physiotherapy. Magnetic resonance imaging (MRI) of the patient’s right shoulder showed supraspinatus tendinopathy with a partial tear. There was a soft tissue mass in the subacromial space measuring 2.5 × 1.0 × 0.5 cm. There were no features of an aggressive lesion or any local infiltration (Figure 1). Following a discussion of the risks and benefits of conservative versus surgical intervention, she decided to undergo an arthroscopic excision biopsy.

At surgery her glenohumeral joint was normal. The subacromial space revealed a soft tissue mass. It was attached to the bursal tissue without any obvious connection to muscle. The mass was excised by marginal excision and subacromial bursectomy was completed.

**Figure 2:**
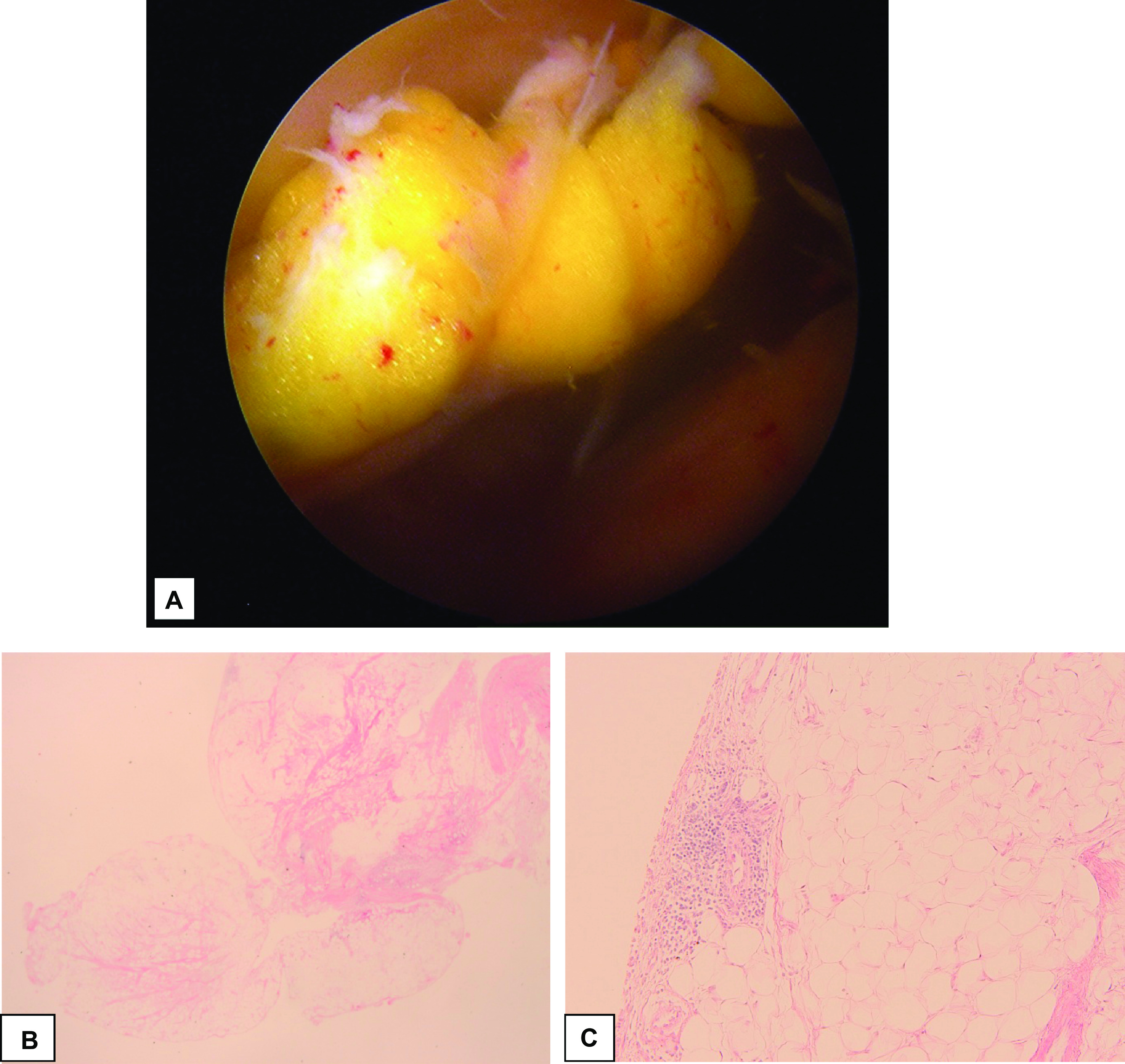
(A) Intra-operative subdeltoid lipomatous mass pre-excision. (2.3 × 1.0 × 0.5 cm). (B and C) Histologic features of lipoma Arborescence: (B). Papillary lipomatous proliferation (C). Lipomatous lesion with surface synovial lining and a focus of lymphoplasmacytic infiltration (H&E stain).

The gross specimen measured 2.3 × 1.0 × 0.5 cm (see Figure 2). Specimens were taken with arthroscopic biopsy forceps and the mass was removed en bloc by use of a synovator. Histology showed a lipomatous lesion with a surface synovial lining and a focus of lymphoplasmacytic infiltration (Figure 2).

At 5 years follow up, the patient had a full active range of movement of her shoulder and normal power of her rotator cuff.

## Discussion

Subacromial impingement syndrome (SIS) is the leading cause of shoulder pain, representing 40–60% of all shoulder pain visits to orthopaedic clinics [1]. As an extrinsic cause of shoulder impingement [2], there are few reported tumours arising from the coracoacromial arch [3, 4]. In addition, limited cases of sub-acromial space-occupying lesions have been reported as a cause of SIS, such as lipoma [5, 6] and lipoma arborescens [7–15].

Moreover, Singh et al. drew attention to lesions outside subacromial space as a cause of subacromial impingement, these include supraspinatus intramuscular lipoma or cysts and supra-scapular glomus tumour [16]. Tumours in the shoulder region such as metastatic carcinoma and myeloma should not be overlooked, as they are not uncommon and can be misdiagnosed as rotator cuff tears [17]. Ogose et al. [18] reported the histologic types of coracoid process tumours as chondrosarcoma, osteosarcoma, osteoid osteoma, giant cell tumor, and aneurysmal bone cyst. Mavrogenis et al. confirmed that chondrosarcomas, osteoblastomas, and chondroblastomas are the most common bone tumors of the coracoid process [19].

Only nine cases of subacromial lipoma arborescens associated with impingement syndrome have been reported in the literature [7–15].

Lipoma arborescens is a benign, diffuse villous proliferation of the synovium characterized by the replacement of the subsynovial tissue by mature adipocytes [20]. The knee is the most common location of lipoma arborescens, however, it has been reported in other joints such as the shoulder, elbow, and hip [21].

Both Sanamandra and Ong [21] and Ryu et al. [22] have shown the efficacy of MRI in the diagnosis of lipoma arborescens. Sanamandra and Ong outlined the MRI features of lipoma arborescens [21]. In our case, MRI showed the typical appearance of a fat-containing, lobulated synovial mass (2.5 × 1.0 × 0.5 cm) in the subacromial-subdeltoid bursa with fat signal intensity on all MRI sequences (Figure 1). Burt and Huang in 2017 reported that the presence of fat within the synovium is diagnostic [20] and all MRI sequences of the lesion have the same signal intensity of fat [20, 22].

Histopathology slides of our patient’s lesion showed papillary lipomatous proliferation on a lower power field. Higher power field showed lipomatous lesion with surface synovial lining and a focus of lymphoplasmacytic infiltration, without atypical adipocytes (H&E stain) (Figures 2A and 2B). Similar results have been reported by Minami et al. when they showed mature lipid cells without malignant features, surrounded by thick synovial folds. The latter contains dense and focally nodular lymphocytic and plasmacellular infiltrate. Liposarcoma could be excluded by the absence of atypical lipoblasts [23].

## Conclusion

Lipoma arborescens represents a rare cause of subacromial impingement. A systemic approach for abnormal causes of SIS is recommended to avoid misdiagnosing rare or sinister pathologies, which may hinder recovery and/or lead to poor results.

In the absence of any red flags in history and examination coupled with a benign appearance on image screening, arthroscopic marginal excision is a safe and effective treatment. Complete bursectomy of the subacromial space may minimize the risk of recurrence.

## Conflict of interest

All authors declare that there was no conflict of interest in conducting this work.

## Patient consent

Written informed consent has been obtained from the patient to publish her information, including image scans.
